# Self-Management in Persons with Limb Loss: A Systematic Review

**DOI:** 10.33137/cpoj.v4i1.35098

**Published:** 2021-06-04

**Authors:** DJ Lee, T Repole, E Taussig, S Edwards, J Misegades, J Guerra, A Lisle

**Keywords:** Self-management, Limb Loss, Prosthesis, Amputation, Systematic Review

## Abstract

**BACKGROUND:**

Self-management is an integral component of managing long-term conditions and diseases. For a person with limb loss, this self-management process involves caring for the residual limb, the prosthesis, and the prosthetic socket-residual limb interface. Failure to properly self-manage can result in unwanted secondary complications such as skin breakdown, falls, or non-use of the prosthesis. However, there is little evidence on what self-management interventions are effective at preventing secondary complications. To understand the impact of self-management after the loss of a limb, it is necessary to determine what the current evidence base supports.

**OBJECTIVES:**

The purpose of this study is to examine the available literature on self-management interventions and/or outcomes for persons with limb loss and describe how it may impact residual limb health or prosthesis use.

**METHODOLOGY:**

A systematic review of multiple databases was carried out using a variety of search terms associated with self-management. The results were reviewed and selected based on the inclusion criteria: self-management interventions or direct outcomes related to self-management, which includes the skin integrity of the residual limb, problem-solving the fit of the prosthesis, and education in the prevention of secondary complications associated with prosthesis use. The Cincinnati Childrens’ LEGEND (Let Evidence Guide Every New Decision) appraisal forms were used to analyze the articles and assign grades.

**FINDINGS:**

Out of the 40 articles identified for possible inclusion in this study, 33 were excluded resulting in seven articles being selected for this review. Three out of the seven articles focused on silicone liner management while the other four articles focused on skin issues.

**CONCLUSIONS:**

Self-management for a person with limb loss is a key component of preventing complications associated with loss of limb and prosthesis use. There is a lack of high-quality experimental studies exploring the most appropriate intervention for teaching self-management when compared to other conditions, specifically diabetes. Further research in the area of self-management is necessary to understand how to best prevent unwanted secondary complications.

## INTRODUCTION

In the United States, approximately 2 million individuals are living with limb loss.^1^ Common etiologies that may lead to amputation include trauma, cancer, and vascular complications secondary to diabetes.^2^ Regardless of the etiology of amputation, living without a limb requires biopsychosocial and behavioral adaptations to successfully reintegrate into society.^3,4^ While biopsychosocial adaptations may be learned through the experience of participating in a comprehensive rehabilitation program,^5^ the behavioral aspects related to lifestyle changes and self-care may go under-emphasized. These behavioral and lifestyle changes for a person with limb loss specifically involve caring for the residual limb and prosthesis, as well as the interface between the two. When considered as a whole, the behavior and lifestyle changes are known as self-management, a term used to describe the daily adaptions one must make when faced with a chronic condition.^6,7^

While a universal definition of self-management after amputation is deficient, it is agreed that optimal self-management requires the patient to be an active participant in decisions regarding their health and own care.^8^ For persons with limb loss, this can be thought of in three distinctive categories: residual limb care and hygiene, problem-solving the fit of the prosthesis, and making decisions regarding self-care.^9^ These decisions surrounding the daily self-care and hygiene associated with residual limb includes washing regimens, liner cleaning and drying, skin inspection, and wound prevention.^10^ Self-management also involves problem-solving the fit of the prosthesis, which may include modification of sock-ply, sequencing components for donning, and maintaining awareness of how the fit changes throughout the day.^11^ Finally, decision-making is concerned with recognizing when an issue presents, such as skin breakdown, and properly contacting the correct medical professional for assistance.^12^

If self-management is not embodied and embraced by persons with limb loss they may be at an increased risk of injury, including but not limited to skin breakdown, wound formation, musculoskeletal issues, or falls.^13-15^ Conversely, proper self-management may be related to better outcomes after loss of limb, including maintaining the integrity of the skin, preventing wound formation, and improving quality of life.^10,16^ Currently, the greatest resources of self-management education are found in clinical textbooks, patient handouts, and through professional organizations. However, despite the importance of self-management, there is a relative paucity of peer-reviewed materials on the subject published in scientific journals specific to which intervention is the most efficacious. While other conditions like diabetes mellitus have well established pathways for assessing and imparting self-management knowledge and behaviors,^17-23^ self-management after limb loss has not received the same level of attention in the scientific literature. To fully comprehend the impact of self-management after loss of limb, it is necessary to determine what the current evidence base supports. Therefore, the purpose of this study is to examine the available published literature on self-management interventions and/or outcomes for persons with limb loss and describe how it may impact residual limb health or prosthesis use.

## METHODOLOGY

### CRITERIA FOR STUDIES CONSIDERED

#### Types of Studies

The study types considered for review were mixed methodologies, retrospective studies, case reports, randomized control trials, and qualitative studies. Participants of any age, gender, background, and limb deficiency were included if self-management was emphasized. Inclusion criteria consisted of studies that assessed functional outcome measures related to self-management, participants of any age or gender, and individuals with upper or lower limb amputation or limb deficiency. Articles published between January 1965 and September 2019 were considered eligible for review. Exclusion criteria were studies published in a language other than English and opinion-based papers. Non-peer reviewed publications (e.g. textbooks, patient education materials) were excluded.

#### Search Strategy

A computerized literature search was conducted from September 2019 to December 2019 by the research team. The search strategy of this review consisted of combining keywords related to the self-management of individuals with limb loss into search permutations. Each permutation was inputted into five databases: PubMed, Cochrane Library, PEDro, Google Scholar and PsycNET. This strategy was developed to locate published studies relevant to individuals with amputations or limb differences and the individual’s ability to self-manage their residual limb or the residual limb-prosthesis interface. Because there are many terms synonymous with limb loss and self-management different combinations of keywords were used. Search keywords are presented in Table 1.

**Table 1:** Search keywords.

Keywords related to “amputee”	Keywords related to “self-management”	
Amputee	Care	Education
Amputation	Caring	Fluid fluctuation
Prosthetic	Self-care	Volume
Prosthetic leg	Self-evaluation	Fluid loss
Prosthesis	Self-managed	Donning
Residual limb	Self-management	Doffing
Limb loss	Self-managing	Socket
	Self-manage	Socket fit
	Evaluation	Prosthetic fit
	Management	
	Problem solve	
	Problem solving	

#### Selection and Rating

**Figure 1:**
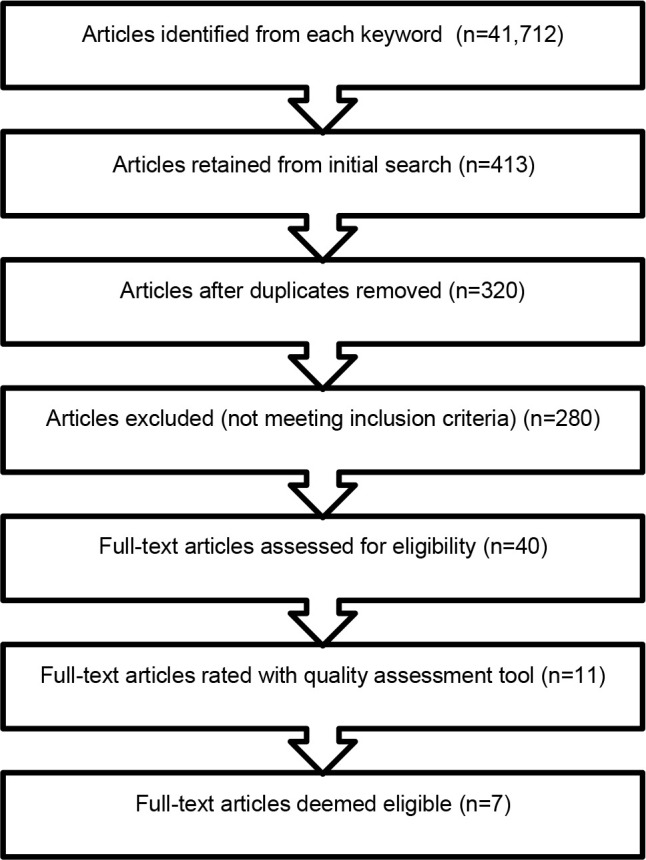
Article eligibility flow chart.

The study eligibility flowchart is presented in Figure 1. The initial search provided numerous results from the keywords used for the search. Many of the results from this initial search were not explicitly related to the topic of self-management in persons with limb loss. Results excluded at this stage were studies that focused on prosthetics for other anatomical structures and health conditions. Article duplicates and non-English studies were then excluded. The remaining 40 articles had an initial face value of being eligible for this systematic review, however upon further examination many focused towards quality of life in those with limb loss rather than self-management of the limb-socket interface. The final remaining 11 articles underwent full evaluation for inclusion.

Given the diversity of study designs represented in the literature, the evaluative tool needed to be flexible enough to account for the variety of results. The Cincinnati Childrens’ LEGEND (Let Evidence Guide Every New Decision) appraisal forms was determined to be the best fit. The LEGEND tool is a series of appraisal forms designed to help clinicians synthesize evidence and determine the quality of published studies of different designs.^24^ The LEGEND scale uses an algorithm to derive a grade of either good or lesser quality, then based on the summative quality of each review an overall score is given base on the types of studies included. Six of the reviewers were put into pairs and each pair was assigned four studies to review. Each reviewer independently assessed the quality of their assigned studies according to a standardized form determined by the study design, and then scores were compared. If there was a disagreement, a third reviewer assessed the study.

## RESULTS

The majority of participants were males (approximate mean age of 61 years) with acquired transtibial level amputations. No randomized control trials including self-management interventions were included. Of the seven included articles, most were of good quality and focused on education and training. Table 2 presents a summary of articles and demographic characteristics. The specific breakdown of each included article can be found in Table 3.

**Table 2:** Summary of the seven included articles and demographic characteristics.

**ARTICLE CHARACTERISTICS**
Historical Cohort	1
Qualitative	2
Case Report	2
Descriptive	2
**QUALITY OF STUDIES**
Good Quality	4
Lesser Quality	3
**SELF-MANAGEMENT**
Impact of Hand Function	1
Efficacy of Education and Training	3
Self-Management Practices	1
Prosthetic Prescriptions	2
**PARTICIPANTS (259 people)**
Males	185
Females	54
Not Disclosed	20
**AGE**
Range	18-90
Approximate Mean Age	61
**LEVEL OF AMPUTATION**
Trans-femoral	33
Knee Disarticulation	13
Trans-tibial	128
Symes	8
Not Disclosed (Includes UE and LE)	77
**TYPE OF LIMB LOSS**
Acquired	250
Congenital	7
Not Disclosed	2

**Table 3:** Overview of studies included in the literature review.

Article title (year)	Number of participants and age (SD)	Level of amputation	LEGEND grade	Article type	Outcomes of interest/intervention	Results
Skin problems of the stump and hand function in lower limb amputations (2008)^25^	n=60(43 M, 17 F) Age: 62.3 (15.4)	TT (50KD (10)	Good Quality (4a)	Historic Cohort Explorative study	Relationship between impaired hand function and liner-related skin problems of the residual limb.	Impaired hand function was significantly related to liner-related skin problems.
The effect of prosthetic rehabilitation in lower limb amputees (1995)^26^	n=29(22 M, 7 F) Age: 64 (n/a)	TT (19)TF (10)	Lesser Quality (4b)	Qualitative (Questionna ire)	Efficacy of training following prosthesis prescription in promoting constant use of the prosthesis.	More effective communication between the patient and healthcare team is needed.
Improper use of a transtibial prosthesis silicone liner causing pressure ulceration (2009)^27^	n=1 (M)Age: 80	TT	Good Quality (5a)	Case Report	Importance of appropriate candidate selection for roll-on liners, proper patient and professional training, and management of patient comorbidities.	Pressure ulcers may be prevented with proper education of caregivers and patients in correct use of all prosthetic components.
Knowledge and skill of patients with regard to amputations stump bandaging, prior to a prosthesis (1998^28^	n=33(21 M, 12 F) Age: 23-78(average age and SD not specified)	LLA	Lesser Quality (4b)	Descriptive Study	Knowledge and skill of patients with regard to residual limb bandaging prior to fitting of a prosthesis. Success post-amputation is highly dependent on the quality of education on residual limb bandaging given.	Success post-amputation is highly dependent on the quality of education on correct residual limb bandaging.
Issues of importance reported by persons with lower limb amputations and prostheses (1999)^29^	n=92(79 M, 13 F) Age: 55 (n/a)	Through the knee, TT, Symes	Good Quality (4a)	Descriptive Study	Improve decisions related to amputation levels and prosthetic prescription.	Fit of the prosthesis socket with the residual limb, aspects of mechanical functioning of the prosthesis, other non-mechanical qualities, and advice about adaptation to life with a prosthesis with support from others are the major themes deemed important to those living with LLA.
Incorporating self-management in prosthetic rehabilitation: case report of an integrated knowledge-to-action process (2015)^30^	n=20 (sex not specified)Age: adult	LLA	Good Quality (5a)	Case Report	Knowledge-to-action process for prosthesis self-management education.	Group training adds value to the prosthesis management education process.
Staying "just normal": preservation strategies in prosthesis use (2019)^31^	n=24(19 M, 5 F) Age: 43.89 (12.66)	Upper and Lower Limb Loss (7 Congenital, 17 acquired,2 not disclosed)	Lesser Quality (4b)	Qualitative Grounded Theory	Practices used by persons with LLA to manage their prosthetic limbs.	Individuals use a variety of preservation strategies to manage threats and limitations of prostheses in order to live “normally” with a prosthesis.

**Abbreviation**: SD, Standard Deviation; n, number; M, Male; F, Female; TT, Trans-tibial; KD, Knee Disarticulation; TF, Trans-femoral; LLA, Lower Limb Amputation

## DISCUSSION

The purpose of this study was to examine the available literature on self-management interventions and/or outcomes for persons with limb loss and describe how it may impact residual limb health and prosthesis use. Despite a systematic search in multiple databases, only seven articles were found to directly address the inclusion criteria of this study.

This is in contrast to diabetes, where a perfunctory search of PubMed using the terms “self-management” and “diabetes” results in thousands of results, including a meta-analysis of systematic reviews.^32^ This brings attention to the lack of self-management-focused research in the limb loss literature as compared to other fields. Since the link between self-management and prevention of secondary complications is well established.^13,33,34^ the lack of strong evidence made apparent by this review limits the ability to provide significant clinical recommendations.

### Review of Studies

When looking at the results of the review it is evident that certain aspects of self-management are promoted regardless of the study, namely the prevention of wound development, proper limb shaping, and contracture prevention. Considering that re-ulceration rates after certain amputations are as high as 70%^35^ it is conceivable that wound prevention is of the utmost importance. Additionally, contracture prevention through exercise and posturing,^36^ as well as limb shaping via bandaging remains a common practice ^37,38^ and should be taught as a basic element of self-management education.

Three of the seven articles ^25,27,29^ directly addressed issues with silicone liners. Silicone liners are commonly used to suspend the prosthetic socket or decrease discomfort from weight-bearing. However, silicone liners have many issues, including creating an inhospitable environment for the residual limb that promotes excessive sweating.^39^ While silicone liners can be used after the initial amputation incision is closed to help shape the limb and decrease the time required for the rehabilitation stay,^40^ they can also result in skin breakdown if donned incorrectly, as exemplified in the case report by Bruno & Kirby.^27^ Therefore, it is of critical importance that proper liner donning be taught as a foundational element of the self-management education process after loss of limb.

Beyond just educating on proper self-management, it may also be necessary to examine the hand strength of the patient as it was shown that those hand impairments are more likely to have skin issues related to liner use.^25^ Considering that older adults have both lower hand strength, more cognitive impairments, and are more likely to experience a lower limb amputation due to a dysvascular condition, the consideration of hand strength in the prescription of a silicone liner should be prioritized.^41,42^

Bandaging remains common practice, however evidence may suggest that the use of rigid removal dressings may have greater benefit.^37,43^ Given the relative ease of donning and doffing, a rigid removable dressing benefits the patient by helping shape the limb, prevent contractures, and protect the limb from environmental impacts.^44^ Despite the benefits of a rigid removable dressing, elastic bandages are still frequently used, likely due to their ubiquitous and affordable nature. Since elastic bandaging comes with inherent risk of injury due to improper donning, education on how to properly apply, remove, and check the fit is necessary^45^ Therefore, proper self-management education on elastic bandage should be performed for any patient using this intervention.

One of the included studies looked at the benefits of group training when learning to self-manage and use a prosthesis.^30^ This practice demonstrated both benefits and drawbacks. The benefits include the ability to socialize, learn from each other, and share in the experience. The drawbacks were that an individual participant in the group setting may offset the benefits based on their actions and beliefs. While self-management can effectively be taught through support groups,^46^ one-on-one educational sessions may still be necessary given the variability in learning styles inherent to learners.

The final study included in this review examined the behaviors and adaptations that need to be made after loss of limb to maintain a state of normalcy.^31^ The behaviors identified in the study (vigilant self-awareness, threat identification, and risk avoidance) are key components of self-managing after loss of a limb. This study identifies that a person with limb loss must be proficient in self-management to maintain a degree of stability and normalcy in their lives. Otherwise, a failure to self-manage can result in a disruption to their established routine, fracturing the state of normalcy that they have re-established since amputation.

### Quality Issues

While the intent of this review was help guide clinical practice in terms of how to best educate the patient on self-management, the paucity of evidence makes this difficult. This is due to the lack of high-quality evidence in the form of randomized control trials and interventional studies. As a result of the lack of primary sources of evidence, lower tier levels of evidence were the only included studies in this review. While descriptive, cohort, qualitative, and case studies are all beneficial in understanding the scope of the current self-management educational interventions, none of the included studies had a comparison group to determine if a specific intervention type was more effective.

### Future Focus

While systematic reviews such as this one brings attention to the need for more research in this area, it makes comparing intervention effectiveness difficult at this junction. As such, there are no explicit interventions for self-management that can be recommended above others. To rectify this situation, future studies are needed to determine if a specific self-management intervention may benefit persons with limb loss more than the current model which depends on clinical expertise, textbooks, and extant patient education material. The field of limb loss rehabilitation could benefit from the model used in study of diabetes which has explored numerous modes of delivering self-management education and their comparative effectiveness. Therefore, funding opportunities should recognize this gap in the literature and support high-quality interventional research studies in order to bolster the body evidence supporting self-management for persons of limb loss.

## CONCLUSION

Self-management is key in the prevention of secondary complications associated with the residual limb, the prosthesis, and the interface between the residual limb and socket. Research needs to be done on appropriate self-management techniques, education, and implementation. Both the patient and their healthcare providers need to be active and engaged throughout the self-management process.

## DECLARATION OF CONFLICTING INTERESTS

The authors have no conflicting interests to disclose.

## AUTHOR CONTRIBUTION

All authors contributed equally in the preparation of this manuscript.

## SOURCES OF SUPPORT

There were no external sources of support to facilitate the completion of this work.

## ETHICAL APPROVAL

Ethical approval was not needed for this study.
